# Supplementary material for: Truncated complement factor H Y402 gene therapy rescues C3 glomerulonephritis
Source: Mol Ther. 2025 Apr 24;33(8):3746–56. doi: 10.1016/j.ymthe.2025.04.035 (PMC12461626; doi:10.1016/j.ymthe.2025.04.035)
Supplement: Supplementary file 1 [file mmc1.pdf]

YMTHE, Volume *33*

Supplemental Information

Truncated complement factor H Y402 gene therapy rescues C3 glomerulonephritis

Lindsey A. Chew, Daniel Grigsby, C. Garren Hester, Joshua Amason, W. Kyle McPherson, Edward J. Flynn III, Meike Visel, Christopher R. Starr, John G. Flannery, Tylor R. Lewis, and Catherine Bowes Rickman

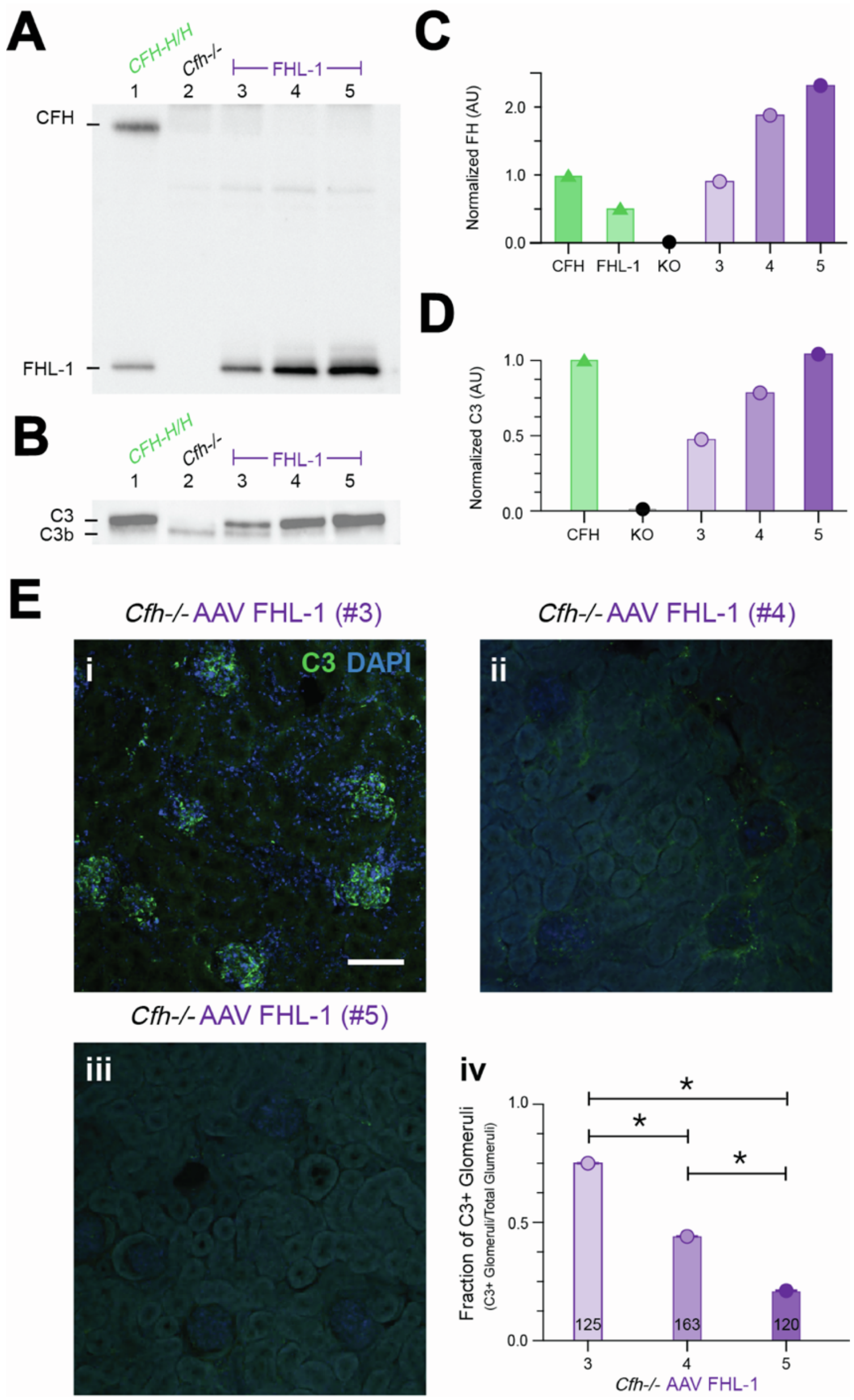

Figure S1. Concentration-dependent, AAV-mediated FHL-1 replacement reduces C3 deposit accumulation and resolves C3G.
(**A**) Western blot analysis of circulating plasma levels of FHL-1 in *Cfh-/-* mice following treatment with AAV-mediated FHL-1 replacement. Each lane shows the corresponding levels of tCFH for a single animal. Lane 1 is a positive control loaded with *CFH H/H* plasma and shows full-length CFH. Lane 2 is a negative control loaded with untreated *Cfh-/-* plasma. (**B**) Western blot analysis of circulating plasma levels of C3/C3b in corresponding *Cfh-/-* mice following treatment with AAV-mediated FHL-1 replacement. Lane 1 is a positive control loaded with *CFH H/H* plasma and shows C3/C3b. Lane 2 is a negative control loaded with untreated *Cfh-/-* plasma and shows C3b but no intact C3. (**C**) Densitometric analysis of Western blot (A) with bar graph (derived from panel A) showing normalized CFH expression in several *Cfh-/-* mice ± AAV-FHL-1 treatment. Normalized FH is calculated as a ratio of FHL-1 expression to endogenous expression of full-length CFH in the CFH-H/H (Lane 1). (**D**) Densitometric analysis of Western

blot with bar graph (derived from panel B) of normalized C3 for each animal and normalized against *CFH-H/H,* C3G-negative control mice. (**E**) Representative immunofluorescent confocal imaging of glomeruli from kidney sections of the corresponding mice (*i-iii*). Scale bar is 100 microns. (*iv*) Bar graph indicating the fraction of C3-positive (C3+) glomeruli out of the total glomeruli for which confocal imaging was obtained. The number of glomeruli analyzed for each cohort is shown at the base of each bar. Unpaired t-test was used for comparison between treatment cohorts, *p<0.01.
