# Supplementary material for: Truncated complement factor H Y402 gene therapy rescues C3 glomerulonephritis
Source: Mol Ther. 2025 Apr 24;33(8):3746–56. doi: 10.1016/j.ymthe.2025.04.035 (PMC12461626; doi:10.1016/j.ymthe.2025.04.035)
Supplement: Supplementary file 2 [file mmc2.pdf]

Molecular Therapy
Original Article

Truncated complement factor H Y402 gene therapy rescues C3 glomerulonephritis

Lindsey A. Chew,[1,5] Daniel Grigsby,[2] C. Garren Hester,[1] Joshua Amason,[1] W. Kyle McPherson,[1] Edward J. Flynn III,[1] Meike Visel,[3] Christopher R. Starr,[4] John G. Flannery,[3] Tylor R. Lewis,[4] and Catherine Bowes Rickman[1,5]

[1]Department of Ophthalmology, Duke Eye Center, Duke University Medical Center, Durham, NC 27710, USA; [2]Genetically Engineered Murine Model (GEMM) Core, University of Virginia, Charlottesville, VA 22903, USA; [3]Department of Molecular & Cell Biology, University of California, Berkeley, Berkeley, CA 94720, USA; [4]Department of Ophthalmology and Visual Sciences, University of Alabama at Birmingham, Birmingham, AL 35294, USA; [5]Department of Cell Biology, Duke University Medical Center, Durham, NC 27710, USA

There are no effective therapies for patients with dry age-related macular degeneration (AMD) or C3 glomerulonephritis (C3G). Unfortunately, past efforts to treat C3G using exogenous human complement factor H (CFH) found limited success due to immune rejection of a foreign protein response. AMD research has also faced myriad challenges, including the absence of an ideal therapeutic target and difficulties with treatment delivery in certain preclinical models. In pursuit of an AMD therapy to overcome these obstacles, we ultimately capitalized on parallels in complement dysregulation between AMD and C3G. Here, we investigate the potential for CFH supplementation as a strategy to rescue C3G. Our findings demonstrate restored inhibition of complement's alternative pathway and long-term reversal of disease without immune rejection using adeno-associated virus (AAV)-mediated delivery of truncated CFH (tCFH) in a *Cfh*$^{-/-}$ mouse model of C3G. We tested three different tCFH vectors and found significant differences in their relative transduction efficiency and therapeutic efficacy. These discoveries motivate the development of AAV-mediated tCFH replacement therapy for patients with C3G while simultaneously demonstrating proof of concept for AAV-mediated tCFH gene augmentation therapy for patients with AMD.

INTRODUCTION

Complement dysregulation is implicated in both age-related macular degeneration (AMD) and C3 glomerulonephritis (C3G). Given the structural, organizational, and functional similarities[1,2] between certain cell types in the eyes and kidneys, pathobiology and pathophysiology shared by both organs may be less surprising. AMD is a global leading cause of irreversible blindness,[3,4] affecting millions of individuals through loss of their high-acuity, central vision. Half of all patients affected by C3G develop end-stage renal failure[5] within a decade of presentation. Patients with C3G are commonly diagnosed with mutations[6–10] in their complement factor H (*CFH*) gene as a primary cause of their kidney disease. These patients also frequently present with coincident ocular subretinal deposits that are indistinguishable[11–13] in their composition from characteristic AMD extracellular deposits known as drusen.[7,14,15] Notably, the increased presence of drusen[16–18] is considered a hallmark of "dry," non-neovascular AMD, and significant AMD risk is conferred by the *CFH* genetic variant[19–23] in which tyrosine is substituted for histidine in amino acid 402 (Y402H). This evidence supports certain shared etiology underlying AMD and C3G while simultaneously highlighting the potential for treatments that rescue both AMD and C3G disease phenotypes. CFH variants and mutations linked to AMD[23] and C3G,[24,25] respectively, motivated our consideration of adeno-associated virus (AAV)-mediated gene therapy as a strategy for treatment of these diseases.

Delivery of AAV-mediated gene therapy to the outer retina remains challenging. Current subretinal injection methods in mouse models are limited by the extensive iatrogenic damage induced by the procedure's requirement for perforation of the neural retina. This obstacle hinders the opportunity for meaningful assessment of visual function in mice following treatment, thereby constraining the development and testing of gene therapy treatments for AMD. However, systemic delivery of AAV-mediated gene therapy by tail vein injection does not face the same challenge. This provides an ideal framework for testing the therapeutic efficacy of an AAV-mediated gene therapy strategy through an alternative model of disease by treating C3G and eliminating renal disease.

We previously published the design of several AAV gene therapy vectors to support human CFH Y402 replacement in *Cfh* knockout (*Cfh*$^{-/-}$) mice,[24,26] which not only provides a straightforward platform for measuring successful gene replacement but also serves as a well-established mouse model of C3G.[24] CFH consists of 20 short consensus repeats (SCRs), with several SCRs known for their roles in C3 or glycosaminoglycan (GAG) binding[27,28] (Figure 1A). We

Received 16 December 2024; accepted 22 April 2025;
https://doi.org/10.1016/j.ymthe.2025.04.035.

Correspondence: Catherine Bowes Rickman, Duke University Medical Center, 5010 Albert Eye Research Institute, 2351 Erwin Road, Durham, NC 27710, USA.
E-mail: bowes007@duke.edu

 © 2025 The Authors.
Published by Elsevier Inc. on behalf of The American Society of Gene and Cell Therapy.
This is an open access article under the CC BY license (http://creativecommons.org/licenses/by/4.0/).

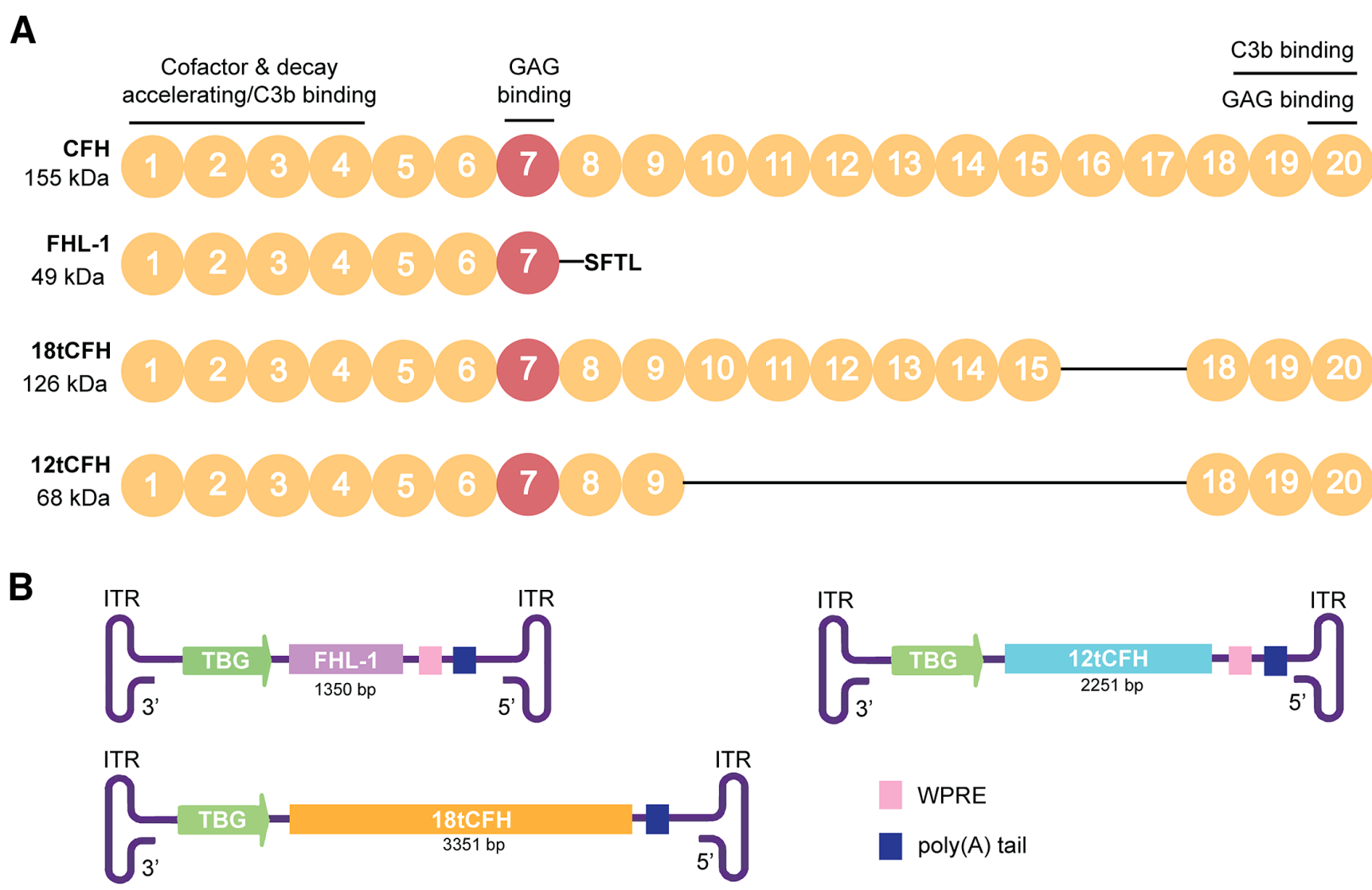

Figure 1. Schematic of CFH and tCFH proteins packaged into AAV-targeting hepatocytes

(A) Full-length CFH consists of 20 short consensus repeats (SCRs), including the SCR spanning the AMD-risk variant CFH Y402H AA (red). Key binding domains and known roles are highlighted. The size (kDa) and retained SCRs for each tCFH protein are also shown. Line connecting SCRs denote missing SCRs compared to CFH. GAG, glycosaminoglycan. (B) Each construct from inverted terminal repeat (ITR) to ITR, with its corresponding promoter (green arrow), *tCFH* cDNA (size in base pairs [bp]). TBG, thyroxine-binding globin; WPRE, woodchuck hepatitis B post-translational response element and poly (A) tail.

designed truncated versions of human CFH and named them according to the total number of SCRs expressed by each truncated CFH (tCFH) cDNA. We initially compared the efficiency of AAVs[26] inducing expression of full-length CFH, 18tCFH, and factor H-like 1 (FHL-1, the endogenous human splice variant of CFH composed of SCRs 1–7) in *Cfh*$^{-/-}$ mice (Figure 1). In untreated *Cfh*$^{-/-}$ mice, CFH deficiency results in complement dysregulation, aberrant C3 tick-over,[24,29,30] and uninhibited consumption of C3 and factor B (FB) by the formation of the C3 convertase, C3bBb, of the alternative pathway of complement (APC).[31] We showed that AAV-mediated CFH Y402 replacement therapy in *Cfh*$^{-/-}$ mice restores complement regulation through the APC, leading to detectable recovery of FB levels.[26] Our findings underscored the importance of local synthesis for ocular complement regulation while simultaneously revealing differences in the efficacy of CFH replacement according to the SCRs expressed following treatment.[26]

In this study, we tested the hypothesis that AAV-mediated human CFH Y402 replacement therapy can be used as a treatment to cure C3G in the *Cfh*$^{-/-}$ mouse model. Previous efforts to treat *Cfh*$^{-/-}$ mice by systemic administration of purified human CFH (hCFH) protein were plagued by long-term immune rejection of the hCFH.[32] In contrast, *CFH*-humanized *Cfh*$^{-/-}$ mice expressing hCFH transgenes do not develop C3G[29,33]; this is true of humanized mice expressing the AMD-protective Y402 variant (*CFH-Y/0*), as well as those expressing the AMD-risk H402 variant (*CFH-H/H*) of hCFH. These mice do not mount a foreign protein response to the hCFH[33] and show that endogenous hCFH can effectively replace mouse Cfh in *Cfh*$^{-/-}$ mice to reverse the C3G phenotype and treat the nephropathy.[2,3] Building on past conceptual achievements in the development of CFH gene therapy,[26,32] this supports the hypothesis that AAV-mediated gene replacement will not induce a foreign protein response like that detected following treatment with exogenous hCFH protein.[32]

Importantly, C3G requires diagnosis by histopathology; more specifically, C3G diagnosis relies on renal biopsy demonstrating evidence of glomerulonephritis[34,35] with sole or dominant glomerular immunofluorescence staining for C3 of at least two orders of magnitude greater intensity than for any other immune reactant. We relied on this gold standard for C3G diagnosis along with histopathological assessment of the kidney glomeruli[10,35] in treated *Cfh*$^{-/-}$ mice to verify successful CFH replacement therapy compared to untreated controls. Our findings provide *in vivo* preclinical evidence for AAV-mediated CFH Y402 replacement therapy as a viable treatment strategy to treat and resolve C3G.

RESULTS

Systemic AAV-mediated expression of hepatic tCFH in *Cfh*$^{-/-}$ mice restores intact C3 and reduces C3b through restoration of the APC in the fluid phase

Complete CFH deficiency in *Cfh*$^{-/-}$ mice leads to uninhibited consumption of C3[24] and cleavage into C3b to support formation of the C3 convertase complex. We hypothesized that AAV-mediated CFH replacement would reduce C3b in the fluid phase by restoring the APC to conserve intact C3. To test this, we designed recombinant AAV constructs optimized for liver expression according to efficiency of the selected AAV serotype and a liver-specific promoter. This design strategy aligned with natural systems where the bulk of systemic CFH is produced by the liver.[36] Specifically, we used AAV8 to efficiently transduce hepatocytes[37] and a thyroxine-binding globin (TBG) promoter[38,39] to drive hepatocyte-specific expression of the tCFH cDNAs. Three tCFH AAVs coding for three truncated versions of CFH were used (Figure 1B). In addition to our previously characterized 18tCFH and FHL-1 AAV expression cassettes, we designed a 12tCFH AAV construct with a strong likelihood of overcoming the limited packaging capacity of AAVs. Containing six fewer SCRs than its 18tCFH counterpart, the 12tCFH AAV construct would be expected to express higher levels of its significantly shorter tCFH gene product.[40] This expectation mirrors observed expression of the FHL-1 construct, which contained 11 fewer SCRs and was expressed at higher levels than the 18tCFH construct.[26] Figure 1A highlights motifs from full-length CFH that are expressed in the tCFH

Table 1. AAV vectors and viral titers

AAV8-TBG-[Vector][a]	Total viral titer delivered (vg) Trial 1	Trial 2	Trial 3
FHL-1	1E12	2E12	1E13
12tCFH	1E12	2E12	1E13
18tCFH	4E12	–	1.5E13

[a]Delivered via tail vein injection in *Cfh*$^{-/-}$ mice.

proteins, including varying relative SCRs with different known functions. Figure 1B depicts the tCFH expression cassettes inserted in AAV8 capsids for use in these studies.

The three AAV8-TBG-tCFH AAVs (Figure 1B) were systemically administered to *Cfh*$^{-/-}$ mice of various ages via tail vein injections. Three trials of this experiment were conducted using the viral titer for each iteration of the experiment as shown in Table 1. Blood was collected 8–12 weeks after tail vein injection from treated animals (*Cfh*$^{-/-}$AAV12tCFH; *Cfh*$^{-/-}$AAVFHL-1; *Cfh*$^{-/-}$AAV18tCFH), untreated *Cfh*$^{-/-}$ animals (which serve as C3G^{+} controls presenting with kidney disease), and healthy *CFH-H/H* animals (which serve as a C3G^{-} control without kidney disease). Plasma was isolated from the blood of each animal. The levels of 12tCFH, 18tCFH, and FHL-1 proteins in the plasma of AAV-treated animals were quantified by western blotting (Figure 2). A comparison of these immunoblots revealed higher levels of circulating 12tCFH and FHL-1 compared to levels of 18tCFH in treated *Cfh*$^{-/-}$ mice (Figures 2A and 2C). We assessed restoration of the APC by quantifying levels of C3 and C3b in the plasma of these same animals (Figures 2B and 2D). While uninhibited C3 tick-over in *Cfh*$^{-/-}$ mice leads to complete consumption of C3, leaving only the cleavage product C3b,[24,29,30] successful AAV-mediated CFH replacement therapy and subsequent restoration of the APC leads to detectable recovery of intact C3 (Figure 2B). The ratio of C3/C3b in each lane provides a normalized scale for restoration of the APC despite animal-to-animal variability in circulating levels of complement and components of its pathways (Figure 2D). Across treated and untreated animals, examining the ratio of C3/C3b in fluid phase (Figure 2D) highlights the concentration-dependent recovery of the APC based on the levels of tCFH or FHL-1 (Figure 2). Although low levels of circulating 18tCFH are observed in *Cfh*$^{-/-}$AAV18tCFH mice, proportional levels of intact C3 are still present (Figure 2).

AAV-mediated CFH replacement reduces C3 deposit accumulation and resolves C3G

Renal biopsy followed by histology and glomerular immunofluorescence are both necessary and sufficient components of C3G diagnosis.[34,35] Previous efforts have characterized the *Cfh*$^{-/-}$ mouse model of C3G by these measures, while simultaneously demonstrating that uncontrolled C3 activation *in vivo* is essential for the development of C3G membranoproliferative glomerulonephritis.[24,29] This requirement is supported by evidence of normal renal function and histology in double-knockout mice (*Cfh*$^{-/-}$*: Bf*$^{-/-}$) with both CFH deficiency and FB deficiency (*Bf*$^{-/-}$), which prevents formation of the C3 convertase C3bBb[24] through the APC. In *Cfh*$^{-/-}$ mice, uninhibited consumption of C3 leads to excesses of C3b/iC3b in the fluid phase, contributing to the accumulation of significant C3b/iC3b deposits in the glomeruli, with subsequent development of kidney disease and, ultimately, renal failure.[32,35,41] We hypothesized that successful tCFH replacement therapy would significantly reduce the accumulation of C3 deposits in treated *Cfh*$^{-/-}$ mice.

To test this, kidneys were harvested for analysis from treated animals (i.e., *Cfh*$^{-/-}$AAV12tCFH; *Cfh*$^{-/-}$AAVFHL-1; *Cfh*$^{-/-}$AAV18tCFH), untreated positive-control (C3G-positive, C3G^{+}) *Cfh*$^{-/-}$ animals, untreated positive-control *CFH-H/0* animals (transgenic mice that are also C3G^{+} because they express full-length hCFH H402 at only ∼10% of wild-type mouse levels[29]), and healthy (C3G^{-}) *CFH-H/H* animals.[33] After sectioning and immunolabeling with anti-C3, we analyzed glomeruli from these kidneys for C3 immunofluorescence (Figure 3A). We conducted a masked analysis of glomeruli from three kidney sections per mouse across 16–24 mice for each *Cfh*$^{-/-}$AAV treatment condition, and we graded each glomerulus as C3^{+} (with C3 immunolabeling present) or C3^{-} (without C3 immunolabeling present). This analysis allowed us to determine the ratio of C3^{+} glomeruli as a fraction of the total number of glomeruli in the sections captured (Figure 3B). Compared to untreated *Cfh*$^{-/-}$ mice, we observed significantly lower fractions of C3^{+} glomeruli (Figure 3B) following AAV-mediated tCFH replacement with 12tCFH and 18tCFH. While AAV-mediated FHL-1 replacement therapy also significantly lowered the C3^{+} glomeruli/total glomeruli ratio, it appeared to be only 50% as effective as 12tCFH or 18tCFH replacement (Figure 3B). Further separation of experimental cohorts treated with FHL-1 replacement revealed a dose-dependent effect of FHL-1 replacement that strongly corresponded with the viral titer administered by tail vein injection (Figures 3Avi, 3Avii, and 3C). The apparent dose-dependent clearance of C3 deposits following treatment by AAV8-TBG-FHL-1 mimics the CFH concentration-dependent effect obtained by the clearance of C3 deposits in glomeruli of *CFH-H/H* mice, but not *CFH-H/0* or *Cfh*$^{-/-}$ mice (Figures 3Aii, 3Aiii, and 3B). In several *Cfh*$^{-/-}$AAVFHL-1 mice exhibiting different plasma concentrations of FHL-1 (Figures S1A and S1C), the recovery of intact C3 (Figures S1B and S1D) corresponds with circulating FHL-1 levels. In the same *Cfh*$^{-/-}$AAVFHL-1 mice, clearance of C3 deposits also occurs in a parallel concentration-dependent pattern (Figure S1D). These findings confirm our previous results,[26] suggesting that 12tCFH and 18tCFH function more efficiently than FHL-1 in plasma (Figure 3B).

We further analyzed this CFH concentration-dependent effect by comparing glomeruli from *Cfh*$^{-/-}$ AAV mice treated across several different doses of total AAV viral genomes (Figure 3C). The fraction of C3^{+} glomeruli was significantly lower in *Cfh*$^{-/-}$AAVFHL-1 mice treated with the highest dose (1E13 vector genomes [vg]) of AAV8-TBG-FHL-1 than in counterparts treated with lower doses of the

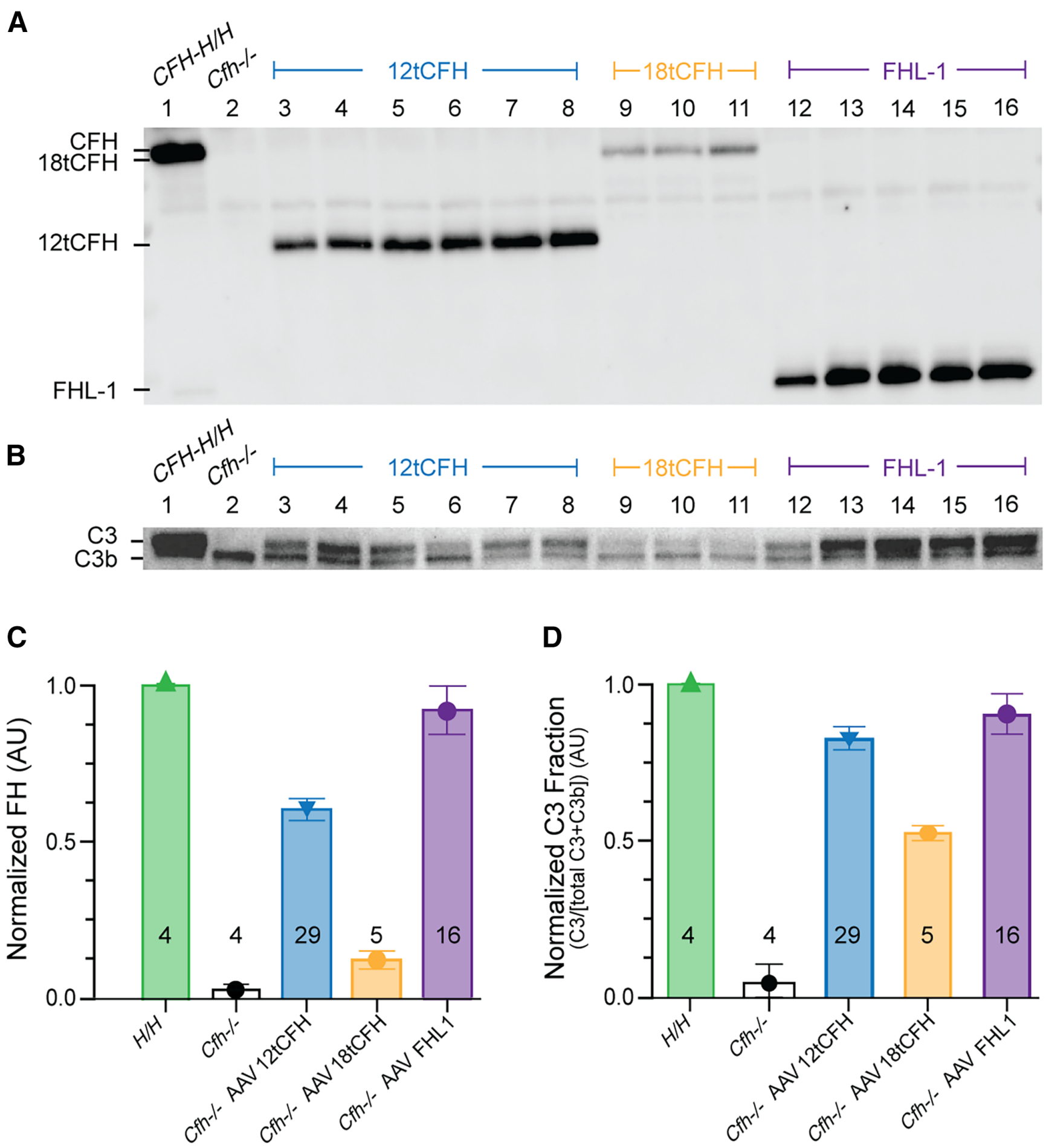

Figure 2. Systemic AAV-mediated replacement of CFH in *Cfh*$^{-/-}$ mice drives hepatic tCFH expression and reduces fluid phase C3b through restoration of the APC

(A) Representative western blot showing circulating plasma levels of 12tCFH (lanes 3–8), 18tCFH (lanes 9–11), and FHL-1 (lanes 12–16) in *Cfh*$^{-/-}$ mice treated by AAV-mediated CFH replacement. Each lane shows the corresponding levels of tCFH for a single animal. Lane 1 is a positive control loaded with *CFH H/H* plasma and shows its endogenous full-length CFH. Lane 2 is a negative control loaded with untreated *Cfh*$^{-/-}$ plasma, which contains no CFH. (B) Representative western blot showing circulating plasma levels of C3/C3b in the corresponding *Cfh*$^{-/-}$ mice treated by AAV-mediated CFH replacement. Each lane shows the corresponding levels of C3/C3b for a single animal. Lane 1 is a positive control loaded with *CFH H/H* plasma and shows C3/C3b. Lane 2 is a negative control loaded with untreated *Cfh*$^{-/-}$ plasma and shows C3b but no intact C3. (C) Densitometric analysis of western blot with bar graph (derived from A) of normalized FH expression in each cohort (±AAV treatment). Normalized FH is calculated as a ratio of tCFH expression to endogenous expression of full-length CFH in the CFH-H/H (lane 1). The number of mice analyzed for each cohort is shown at the base of each bar. (D) Densitometric analysis of western blot with bar graph (derived from B of normalized C3 fraction calculated as the C3 fraction of the total C3 + C3b for each animal and normalized against *CFH-H/H*, C3G^{-} control mice. The number of mice analyzed for each cohort is shown at the base of each bar.

same AAV (i.e., 1E12 or 2E12 vg) (Figure 3C). In *Cfh*$^{-/-}$AAV12tCFH-treated mice, there was no significant difference in the fraction of C3^{+} glomeruli across several cohorts that received different doses. These findings also support a concentration-dependent effect governing clearance of C3 deposits by FHL-1. Lower doses of AAVFHL-1 did not consistently resolve the C3G, while 12tCFH-mediated clearance of C3 deposits does not appear constrained by the same concentration-dependent limitation at the titers tested.

AAV-mediated CFH replacement reduces hallmark histopathological findings in glomeruli and resolves C3G

In the *Cfh*$^{-/-}$ mouse model of C3G, thickening of the glomerular basement membrane (GBM) and mesangial matrix expansion are hallmark characteristics attributed to uncontrolled C3 activation.[24] As a result, we expected tCFH replacement to significantly limit the severity of histopathological findings in the glomeruli of treated *Cfh*$^{-/-}$ mice; specifically, we anticipated that GBM thickness and mesangial matrix expansion would not be significantly different from *CFH-H/H* counterparts without renal disease.

Kidney sections were stained with periodic acid-Schiff (PAS) to highlight any glomerular damage in treated animals (i.e., *Cfh*$^{-/-}$AAV12tCFH; *Cfh*$^{-/-}$AAVFHL-1; *Cfh*$^{-/-}$AAV18tCFH), untreated *Cfh*$^{-/-}$ animals, untreated *CFH-H/0* animals, and healthy *CFH-H/H* animals. Masked graders reviewed the histological appearance of each glomerulus as previously described[29] and assigned a grade to each animal, as shown in Table 2. These results highlight the significant reduction of C3G's hallmark pathologic features in glomeruli of AAV-treated cohorts. Histopathological grades of the treated *Cfh*$^{-/-}$AAV cohorts were significantly lower than the grades of untreated control *Cfh*$^{-/-}$ mice (Figure 4). Hallmark pathologic features such as GBM thickening and extracellular matrix expansion were visible in the untreated *Cfh*$^{-/-}$, *CFH-H/0* (Figures 4Ai and 4Aii) but absent in normal *CFH-H/H* mice (Figure 4Aiii). The pathological features detected in the mice with C3G appear to be resolved or significantly less severe in the *Cfh*$^{-/-}$AAV-treated animals (Figures 4Aiv–4Avi). Comparing the average histopathology grade of each *Cfh*$^{-/-}$AAV treatment cohort reinforces the capacity of AAV-mediated human tCFH expression to significantly reduce glomerular damage. Despite differences in the circulating quantities of CFH, 12tCFH, 18tCFH, or FHL-1, mice across all *Cfh*$^{-/-}$AAV

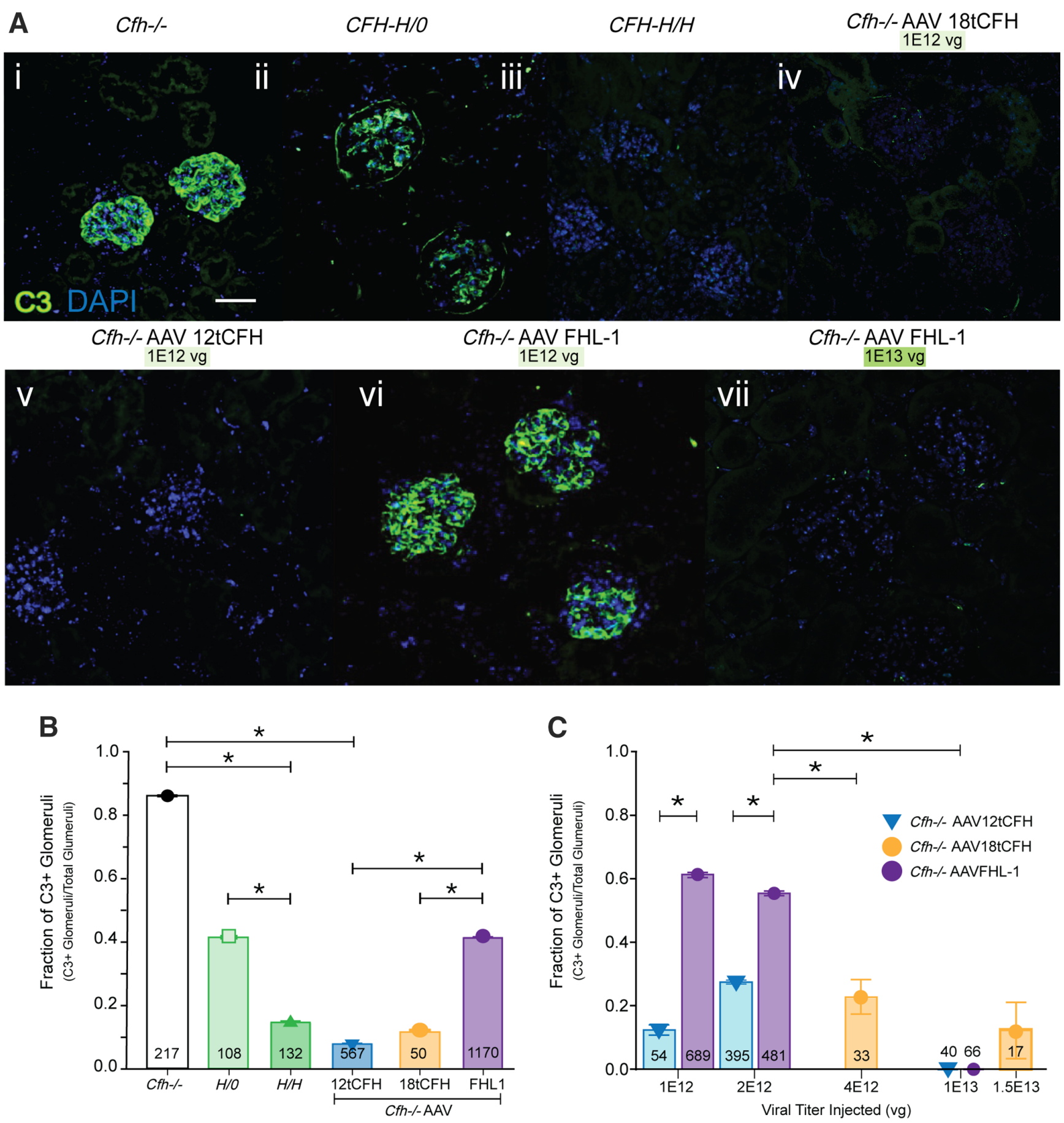

Figure 3. AAV-mediated CFH replacement reduces C3 deposit accumulation and resolves C3G

(A) Representative immunofluorescent confocal imaging of glomeruli from kidney sections of mice according to genotype indicated and AAV treatment (viral titer delivered) as shown. Scale bar, 100 μm. (B) Bar graph indicating the fraction of C3+ glomeruli out of the total glomeruli for which confocal imaging was obtained. The number of glomeruli analyzed for each cohort is shown at the base of each bar. *Cfh*$^{-/-}$ n(mice) = 9; *CFH-H/0* n = 6; *CFH-H/H* n = 9; *Cfh*$^{-/-}$AAV12tCFH n = 29; *Cfh*$^{-/-}$ AAV18tCFH n = 9; and *Cfh*$^{-/-}$AAVFHL-1 n = 16. Unpaired t test was used for comparison between treatment cohorts; *p < 0.01. (C) Bar graph analyzing only AAV-treated *Cfh*$^{-/-}$ mice and assessing treatment as a function of the fraction of C3+ glomeruli out of the total glomeruli imaged. The number of glomeruli analyzed for each cohort is shown at the base of each bar. *Cfh*$^{-/-}$ AAV12tCFH 1E12 vg n(mice) = 5; *Cfh*$^{-/-}$ AAVFHL-1 1E12 vg n = 7; *Cfh*$^{-/-}$ AAV12tCFH 2E12 vg n = 8; *Cfh*$^{-/-}$ AAVFHL-1 2E12 vg n = 4; *Cfh*$^{-/-}$ AAV18tCFH 4E12 vg n = 4; *Cfh*$^{-/-}$ AAV12tCFH 1E13 vg n = 14; *Cfh*$^{-/-}$ AAVFHL-1 1E13 vg n = 5; and *Cfh*$^{-/-}$ AAV18tCFH 1.5E13 vg n = 5. Unpaired t test was used for comparison between treatment cohorts; *p < 0.01.

treatment cohorts exhibited significantly less GBM thickening or extracellular matrix expansion in their glomeruli compared to their untreated *Cfh*$^{-/-}$ and *CFH-H/0* counterparts. The reversal of hallmark, pathologic signs of C3G through AAV-mediated tCFH replacement therapy emphasizes the viability of this strategy to resolve C3G in the *Cfh*$^{-/-}$ mouse.

AAV-mediated CFH replacement resolves hallmark ultrastructural findings of C3G pathology

Previous studies[24,29] have also demonstrated that the *Cfh*$^{-/-}$ mouse exhibits hallmark ultrastructural findings consistent with C3G. These signs include observations of GBM thickening and mesangial matrix expansion under electron microscopy. We anticipated that tCFH replacement would also resolve ultrastructural signs of C3G in treated *Cfh*$^{-/-}$ mice.

Ultrastructural changes in *Cfh*$^{-/-}$ glomeruli were analyzed by transmission electron microscopy. We examined kidney sections from untreated *Cfh*$^{-/-}$ animals, healthy *CFH-H/H* animals, and treated animals (i.e., *Cfh*$^{-/-}$AAV12tCFH; *Cfh*$^{-/-}$AAVFHL-1; *Cfh*$^{-/-}$AAV 18tCFH). We focused on the relative thickness and organization of the GBM and the mesangial matrix. We detected GBM thickening, mesangial matrix expansion, and mesangial deposits in the glomeruli of untreated *Cfh*$^{-/-}$ mice (Figure 5A) consistent with the gross pathological changes that were observed through light microscopy following PAS staining (Figure 4I). These findings are absent in the healthy *CFH-H/H* mouse (Figure 5B). In electron micrographs from AAV-tCFH-treated animals, we also observed a marked reduction in GBM thickening and the absence of mesangial deposits (Figures 5C–5E). These results were consistent with findings from light microscopy following PAS staining (Figures 4iv–4vi). The electron microscopy findings further establish the ability of AAV-mediated tCFH replacement therapy to reverse characteristic signs of C3G and to resolve C3G in the *Cfh*$^{-/-}$ mouse.

DISCUSSION

C3G develops from uncontrolled C3 activation resulting from CFH deficiency in *Cfh*$^{-/-}$ mice and certain patients.[24] CFH deficiency can arise from loss-of-function mutations and truncations,[42,43] from inadequate CFH expression,[44] or even from failure of CFH-associated transcription factors,[45,46] and these failures ultimately result in the dysregulation of C3 tick-over.[24,29,30] Although past efforts to restore CFH in the circulation through supplementation with exogenous hCFH[32] were stymied by the activation of anti-human CFH antibodies in the *Cfh*$^{-/-}$ mouse, our findings clearly demonstrate the feasibility of an AAV-mediated gene therapy for CFH replacement to treat C3G. Since the *Cfh*$^{-/-}$ mouse is well established as a model of C3G,[24] reversing disease in this mouse is an important

Table 2. Grading of mouse glomeruli histopathology in each treatment cohort

Cfh$^{-/-}$		*CFH-H/0*		*CFH-H/H*		*Cfh*$^{-/-}$ AAV12tCFH		*Cfh*$^{-/-}$ AAV18tCFH		*Cfh*$^{-/-}$ AAVFHL-1	
ID	Grade[a]	ID	Grade	ID	Grade	ID	Grade	ID	Grade	ID	Grade
170	3	393	2	007	0	2954	0	2971	2	2966	1
390	3	007	2	008	0	2955	0	2972	2	2967	0
				22	1	2957	1	2973	2	2968	2
				23	0	2958	1	2974	0		
						2959	1	2975	1		
						2960	1				
						2961	1				
						2962	2				
						2964	1				
						2965	1				

[a]In each treatment cohort, images of mouse glomerulus histopathology were graded according to the procedure described in the materials and methods. Grades were assigned to each animal according to the average severity of its glomerular histopathology. The most severe histopathology was found in *Cfh*$^{-/-}$ (C3G$^+$ controls) averaging grade 3, while *CFH-H/H* (C3G-controls) averaged grade 0.

step in the development of a cure for C3G. More specifically, measurable pathologic features of the *Cfh*$^{-/-}$ mouse include glomerular C3 deposit accumulation (Figure 3), GBM thickening, and mesangial matrix expansion (Figures 4 and 5); quantifiably reversing these endpoints following successful treatment strongly suggests progress toward an effective C3G therapy. We show that treatment with each of our AAV vectors (AAV8-TBG-12tCFH, AAV8-TBG-18tCFH, and AAV8-TBG-FHL1) could successfully rescue C3G in this model. Evidence from not only the plasma of treated animals (Figure 2) but also the glomeruli of treatment animals underscored the dramatic phenotypic change with treatment (Figures 3 and 4). Successful treatment through this AAV-mediated strategy also avoided long-term immune rejection and systemic damage, with no evidence of immune infiltration and rejection in the kidneys (Figure 4), in contrast to the previously described failure of treatments injecting exogenous hCFH into *Cfh*$^{-/-}$ mice. Specifically, the treatment of C3G in *Cfh*$^{-/-}$ mice with exogenous hCFH for more than 5 days led to glomerular reactivity with anti-C3 antibodies, due to the deposition of mouse immunoglobulin G (IgG)-hCFH immune complexes.[32] Our 12-week treatment regimen did not show these staining patterns. A likely explanation for success and evasion of immune rejection in this strategy includes that an AAV-mediated strategy directing liver-specific expression of tCFH constructs allows for endogenous glycosylation of the gene product. In contrast, exogenous introduction of injected hCFH[32] includes species-specific exogenous glycosylation that is more likely to flag the immune system against the foreign gene product. Furthermore, evidence suggests that liver-directed AAV gene therapy can induce systemic tolerance to a transgene,[47,48] and a liver-directed strategy likely provides additional support to the successful evasion of immune rejection observed here.

However, the AAV treatments were not equivalent in their efficacy. In the *Cfh*$^{-/-}$AAV18tCFH cohort, treatment by AAV8-TBG-18tCFH did not induce 18tCFH expression levels as high as 12tCFH and FHL-1 levels achieved by the AAV8-TBG-12tCFH and AAV8-TBG-FHL1 vectors. The 18tCFH only has two fewer SCRs than full-length CFH, and its corresponding cDNA sequences reflect this size difference. Limitations in AAV packaging capacity likely constrain expression of the 18tCFH compared to its substantially smaller 12tCFH and FHL-1 counterparts.[26,40] Therapeutic efficacy of the AAV-mediated tCFH treatment strategies depends on sufficient systemic levels of 18tCFH to prevent uncontrolled C3 activation. Insufficient expression of full-length CFH or any tCFH would not sufficiently dampen uncontrolled C3 activation, enabling subsequent accumulation of the C3 by-products in the glomeruli. Previous observations of the *CFH-H/0* mouse (C3G$^+$) compared to the *CFH-H/H* mouse (C3G$^-$) confirm this pathophysiology, with CFH insufficiency facilitating the development of C3G pathology in the *CFH-H/0* mouse.[29] Without enough CFH or tCFH, C3G and the diseased renal phenotype remain.

Significant differences in the therapeutic efficacy of 12tCFH and FHL-1 underscore the importance of C terminus SCRs 18–20 in facilitating C3b and GAG binding. Evidence suggests that efficient GAG binding to cell surfaces primarily depends on SCRs 18–20,[49] and this likely contributes to the divergence in 12tCFH and FHL-1's respective capacities to facilitate C3 clearance in the glomeruli. The absence of critical domains on SCRs 18–20 likely contributes to reduced C3b and GAG binding of FHL-1. Decreases in FHL-1-mediated surface binding and inhibition of C3 activation may also translate to requirements for higher viral titers to achieve therapeutic expression levels. This aligns with previous studies showing that absence of the full length of the CFH C terminus prevents surface recognition[50] functions of the molecule, and therefore likely moderates the surface-recognition capacity of tCFH as well. Accordingly, requirements for higher viral titers facilitating treatment can be reviewed in Figure 3 by comparing *Cfh*$^{-/-}$AAV12tCFH to

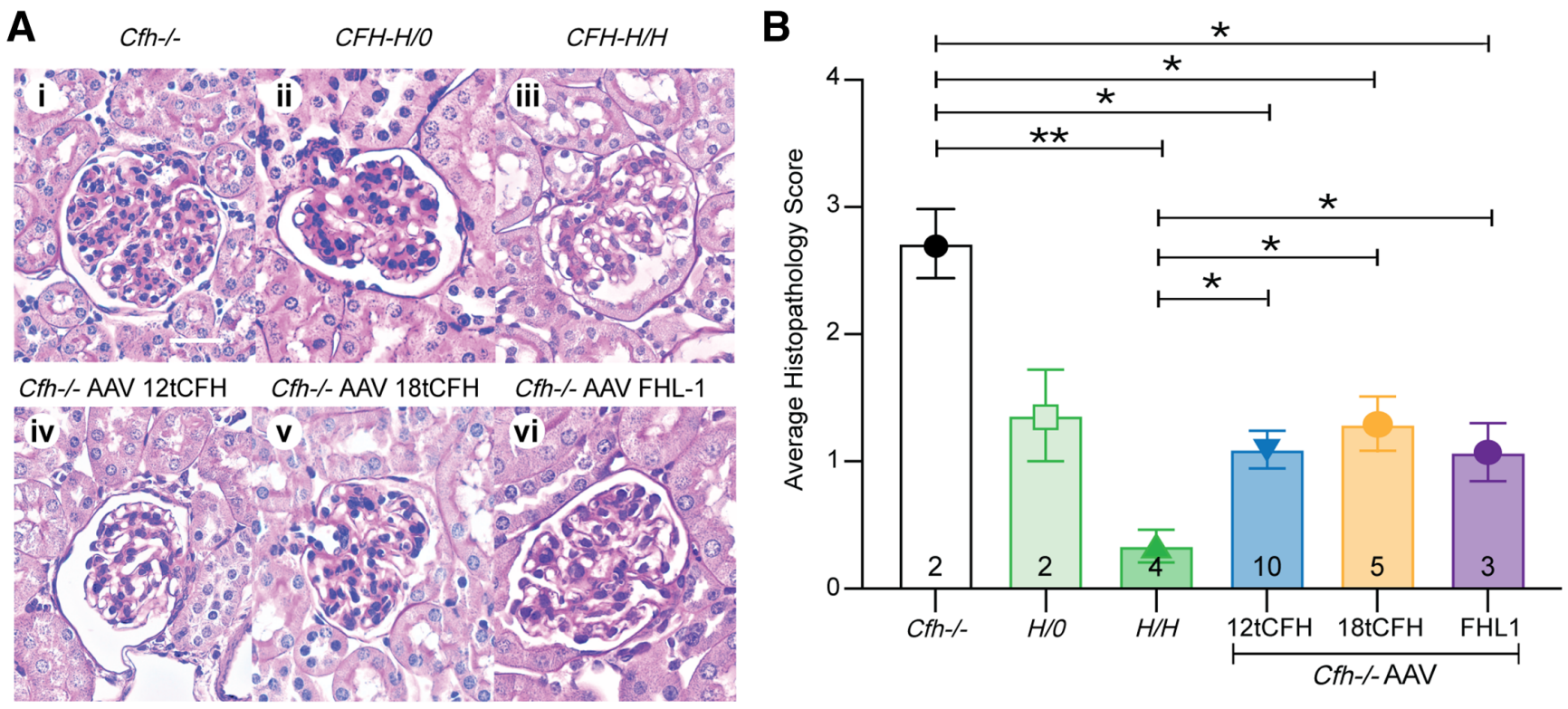

Figure 4. AAV-mediated CFH replacement minimizes hallmark histopathological findings in glomeruli and rescues C3G

(A) Representative images following immunohistochemistry using periodic acid-Schiff staining to highlight features of the glomerulus of a kidney. Scale bar corresponds to 20 μm. (i) $Cfh^{-/-}$ glomeruli had notable hypercellularity, some mesangial matrix expansion, and basement membrane (BM) thickening. (ii) *CFH-H/0* mild hypercellularity, mild BM thickening, mild mesangial matrix expansion. (iii) *CFH-H/H* healthy glomeruli. (iv) $Cfh^{-/-}$AAV12tCFH (1E13 vg), healthy glomeruli. (v) $Cfh^{-/-}$AAV18tCFH (1.5E13 vg), hypercellularity and mesangial matrix expansion. (vi) $Cfh^{-/-}$AAVFHL-1 (1E13 vg), mild hypercellularity, and mild mesangial matrix expansion. (B) Bar graph comparing masked grades assessing histopathology across mice with and without treatment. The number of mice in each cohort is shown at the base of each bar. Unpaired t test was used for comparison between treatment cohorts; $*p < 0.05$ and $**p < 0.01$.

$Cfh^{-/-}$AAVFHL-1 rescue. Analysis of C3G pathology and C3 immunofluorescence in these animals further supports the likelihood of distinct mechanisms underlying the efficacy of full-length CFH or tCFH compared to FHL-1. This is reinforced by focused analysis to examine several $Cfh^{-/-}$AAVFHL-1 mice exhibiting a range of circulating plasma FHL-1 levels (Figures S1A and S1C). In these mice, lower plasma FHL-1 levels corresponded with relatively low recovery of intact C3 (Figures S1B and S1D) and relatively high C3 immunolabeling of their glomeruli (Figures S1Ei and S1Eiv); higher plasma FHL-1 levels matched with relatively high recovery of intact C3 (Figure S1) and relatively low C3 immunolabeling of their glomeruli (Figures S1Eii–S1Eiv). These findings indicate that high doses of FHL-1 may be capable of rescuing C3G, even if this rescue is less efficient than that mediated by 12tCFH.

Interestingly, the localized therapeutic potential of FHL-1 for rescuing C3G appears to be at odds with previous findings highlighting the inability of FHL-1 to restore ocular complement regulation[26] following subretinal injection. Initially, structural similarities between the eye and kidney (i.e., Bruch's membrane in the eye and the GBM both contain a network of α3, α4, and α5 type IV collagen chains[51,52]) suggest that both tissues would respond similarly following treatment with AAV8-TBG-FHL-1. However, the treatment of C3G depends on restoration of the APC in the fluid phase, while the treatment of AMD pathology requires a localized response and binding in Bruch's membrane. For example, significant accumulation of high-density lipoproteins in Bruch's membrane contribute to AMD,[33] and clearance of these deposits may require surface binding recognition from a therapeutic molecule (i.e., 12tCFH, 18tCFH) that outcompetes and ultimately displaces the high-density lipoproteins.[53] In contrast, C3G pathology likely results from uncontrolled C3 activation and its cleavage into C3b, which devolves into excesses of C3b in the fluid phase that are ultimately converted to iC3b[41,54] before accumulating in the glomeruli; this pathophysiology inherently imposes fewer requirements for surface recognition binding. This interpretation of our findings and of exogenous 12tCFH and FHL-1's therapeutic capacities direct our consideration of 12tCFH as a top candidate for follow-up studies to develop treatments for C3G. This study provides critical evidence to suggest that AAV-mediated expression of 12tCFH may be sufficient to restore the APC, eliminate C3 deposit accumulation, and resolve the disease burden of C3G.

Our data also support the high therapeutic potential for the application of an AAV-mediated tCFH Y402 augmentation strategy for the treatment of dry AMD. Strong evidence indicates that the H402 variant of CFH confers significant risk of AMD,[14,23,33] but it remains to be seen whether a tCFH Y402 gene augmentation could successfully rescue an AMD-like phenotype. Future studies to assess this possibility in a mouse model of AMD[33] will be invaluable to our understanding of complement dysregulation in AMD and to the identification of novel treatments. In particular, the capacity of tCFH to clear C3 deposits from glomeruli in C3G suggests that tCFH may be capable of clearing pro-inflammatory accumulates (i.e., high-density lipoproteins,[53] C-reactive protein,[55] and other accumulated molecules) from Bruch's membrane in AMD. Our data introduce the exciting possibility of AAV-mediated CFH augmentation or replacement as therapeutic strategies, and we establish an important foundation for subsequent efforts to develop novel treatments for patients with C3G and AMD, respectively.

MATERIALS AND METHODS

Generation of plasmids and AAV constructs

The 18tCFH cDNA plasmid codes for the 20 SCRs from full-length CFH except SCRs 16 and 17 (Figure 1A) and was sequence verified. The 12tCFH cDNA encodes SCRs 1–9 and 18–20 (Figure 1A) and was sequence verified. The 18tCFH, 12tCFH, and FHL-1 cDNAs code for the normal Y402 amino acid tyrosine at position 402 (Y402). The TBG-FHL-1-woodchuck hepatitis virus post-transcriptional regulatory element and TBG-18tCFH plasmids with flanking AAV inverted terminal repeat (ITR) sequences were generated by VectorBuilder (Chicago, IL) (Figure 1B).

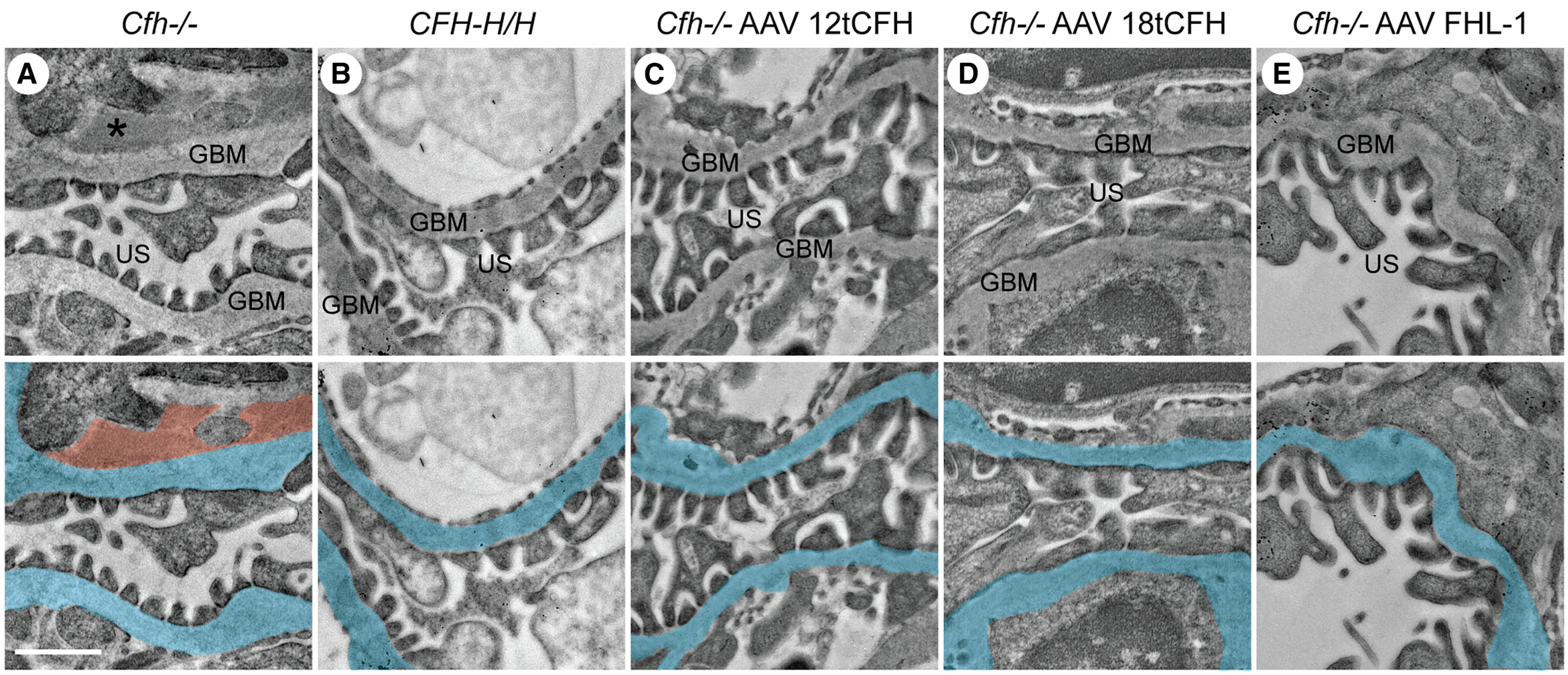

Figure 5. AAV-mediated CFH replacement resolves hallmark ultrastructural findings of C3G pathology

Representative transmission electron micrographs highlighting ultrastructural features of the GBM and mesangial matrix within glomeruli under treated and untreated conditions. Scale bar corresponds to 1 μm. Pseudo-color at bottom highlighting GBM (blue) and mesangial deposits (orange). (A) $Cfh^{-/-}$ ($C3G^{+}$) showing GBM thickening and mesangial deposits (*). (B) *CFH-H/H* (healthy, $C3G^{-}$) showing normal GBM thickness without mesangial deposits. (C–E) $Cfh^{-/-}$AAV12tCFH (1E13 vg), $Cfh^{-/-}$AAV18tCFH (1.5E13 vg), and $Cfh^{-/-}$AAVFHL-1 (1E13 vg), showing mild GBM thickening without mesangial deposits in treated glomeruli. GBM, glomerular basement membrane; US, urinary space.

The plasmids were packaged into AAV8 capsids as previously described.[56] Briefly, recombinant AAV was packaged and purified as previously described.[56] The serotype plasmid AAV8, the transgene containing plasmid flanked by viral ITRs (e.g., 18tCFH, FHL-1, and 12tCFH transgenes), and the adenoviral helper plasmid pHelper were transfected in 293T cells (ATCC CRL-3216) using 1 mg/mL PEI MAX. At 72 h after transfection, cells were harvested and lysed and the virus was purified using an iodixanol ultracentrifugation gradient. An Amicon Ultra-4 Centrifugal filter unit with a 100-kDa molecular weight cutoff was used for buffer exchange and concentration in sterile PBS. The final viral genome titer was measured by qPCR using primers for the ITRs.

Animals and viral injections

All mice were housed conventionally under ambient light conditions to control for environmental factors and microbiome fluctuations. Mice were maintained in accordance with the Institutional Animal Care and Use Committee at Duke University. The number of mice used for each experiment is provided in the figures or figure legends. $Cfh^{-/-}$ mice[24,29] were generated by Dr. Marina Botto. *CFH-H*, *CFH-H/H*, and *CFH-Y/0* mice were generated, characterized, and genotyped as previously described.[29] Only male mice were used in this study. This is based on the original characterization of the $Cfh^{+/-}$ mouse in the Botto lab[24] and our own studies[31] showing that $Cfh^{+/-}$ male mice expressed 50% of wild-type mouse levels, whereas $Cfh^{+/-}$ female mice expressed 80% of wild-type mouse levels. These past results suggested that male $Cfh^{-/-}$ mice would be likely to exhibit the most severe C3G pathology and simultaneously yield the greatest opportunity for therapeutic rescue.

For lateral tail vein injections, mice were restrained in a Tailveiner restrainer device (catalog no. TV-150, Braintree Scientific, Braintree, MA), and tail veins were dilated by contact with a SnuggleSafe heating pad (catalog no. 6250, Lenric C21, Littlehampton, UK). Then, 100 μL of solution containing FHL-1, 18tCFH, or 12tCFH (vg according to each figure) suspended in sterile saline was delivered via a Micro-Fine 28G insulin syringe (catalog no. 329424; Becton Dickinson, Franklin Lakes, NJ).

Western blots

Twelve weeks following tail vein injection, mice were euthanized with CO_2, and samples collected[29,34,35] following transcardial perfusion with phosphate-buffered saline (PBS). Samples were prepared and western blots run as we have previously described.[26,33] Briefly, blood was collected via submandibular vein bleeds in tubes containing EDTA (BD Microtainer, BD Biosciences, Franklin Lakes, NJ) and spun at 4°C to collect plasma. Euthanasia by CO_2 and transcardial perfusion followed. Plasma samples were diluted in a 10% 4× stock of XT Sample Buffer (catalog no. 1610791; Bio-Rad, Hercules, CA), with equal volume dilutions corresponding to equal protein quantification. Western blots were run with non-reducing conditions using precast 10% Criterion XT Bis-Tris polyacrylamide gels (catalog no. 3450112, Bio-Rad) and MOPS buffer (catalog no. 1610788, Bio-Rad), with an equal volume of diluted plasma per plasma sample. After transfer, the nitrocellulose membranes were blocked with 10% bovine serum albumin or milk and incubated overnight with primary antibodies: goat anti-CFH (1:10,000; catalog no. A312, Quidel, San Diego, CA), goat anti-FB (Kent Laboratories, Bellingham, WA), and rabbit anti-C3 (1:5,000; catalog no. CSB-PA365507LA01MO,

Cusabio, Houston, TX). Washes were done with Tris-buffered saline with 0.1% Tween 20 detergent. Incubation with peroxidase-conjugated bovine anti-goat secondaries (catalog no. 805-035-180, Jackson ImmunoResearch Labs, West Grove, PA) for 1–2 h was followed by chemiluminescent detection by Pierce ECL (catalog no. 32132; Thermo Fisher Scientific, Waltham, MA) with a ChemiDoc Imaging System (Bio-Rad). Densitometry was performed using ImageJ software with background subtraction for each lane (National Institutes of Health, Bethesda, MD). Normalization calculations were performed by scaling to expression levels in *CFH-H/H* mice.

Immunofluorescence and confocal microscopy

Mice were euthanized using CO_2 and were perfused transcardially with 30 mL PBS, followed by 4% paraformaldehyde in 0.1 mol/L phosphate buffer, pH 7.4. Kidneys were postfixed in the same fixative overnight, and sections were cut using a vibratome into 50-μm-thick sections. Free-floating sections were placed into glass shell vials and blocked with 0.5 mL 10 mM EDTA/0.1% Triton X-100/10% normal donkey serum in PBS and incubated for 1 h at room temperature. The diluent was removed, and the sections were washed with PBS. A total of 0.5 mL goat anti-mouse C3 antiserum (catalog no. 55444, MP Biomedicals, Santa Ana, CA) at a dilution of 1:500 in PBS was added, and the sections were incubated overnight at 4°C while rocking (Belly Dancer Orbital Shaker, catalog no. BDRAA115S, IBI Scientific, Dubuque, IA). The diluent was removed, followed by 3 washes in PBS. We added 0.5 mL of the secondary antibody Alexa Fluor TM 488 conjugated donkey anti-goat IgG (H + L) (catalog no. A11055, Invitrogen, Carlsbad, CA) diluted 1:500 in PBS and incubated for 90 min at room temperature while rocking. After multiple washes, DAPI (catalog no. 62248, Thermo Fisher Scientific) diluted 1:1,000 was added to counterstain the nuclei. Images were acquired using a Nikon AXR laser scanning confocal microscope (Nikon Instruments, Tokyo, Japan).

Glomerular immunofluorescence was graded according to whether $C3^+$ immunofluorescent labeling was observed on a given glomerulus. All the glomeruli within an image were counted, and the $C3^+$ fraction of glomeruli for that image was recorded. Overall, 5–15 glomeruli per image (8–12 images per mouse) were analyzed by masked investigators.

PAS staining and light microscopy

Kidneys from perfused mice were postfixed overnight in the same fixative before being dehydrated and embedded in paraffin. We cut 1-μm sections and stained them with PAS reagent. Glomerular histologic appearance was graded using the grading system described previously[24] in which grade 0 was normal; grade I, segmental hypercellularity in 10%–25% of the glomeruli; grade II, hypercellularity involving >50% of the glomerular tuft in >25%–50% of the glomeruli; grade III, hypercellularity involving >50% of the glomerular tuft in >50%–75% of the glomeruli; and grade IV (the most severe), glomerular hypercellularity in >75% or crescents in >25% of the glomeruli. For grading, 2–3 glomeruli per image (18–24 images per mouse) were graded by masked investigators (L.A.C. and C.G.H).

Transmission electron microscopy

Kidneys from perfused mice were postfixed in 2% paraformaldehyde in PBS overnight. Tissues were embedded in 2.5% low-melt agarose (catalog no. VF-AGT-VM, Precisionary, Greenville, NC) and sectioned into 200 μm thick sections on a Vibratome (catalog no. VT1200S, Leica, Buffalo Grove, IL). Agarose sections were stained with 1% tannic acid (catalog no. 21700, Electron Microscopy Sciences, Hatfield, PA) and 1% uranyl acetate (catalog no. 22400, Electron Microscopy Sciences), followed by ethanol dehydration and embedding in Spurr's resin (catalog no. 14300, Electron Microscopy Sciences). Sectioning and imaging were performed in the University of Alabama at Birmingham High Resolution Imaging Facility using a JEM-1400 transmission electron microscope (JEOL, Peabody, MA) at 120 kV with a NanoSprint43 MK-II digital camera (AMT, Woburn, MA). Image analysis and processing were performed with ImageJ.

Statistical analysis

Data were plotted as individual points, with error bars representing the standard error of the mean. Unpaired t tests were used for single comparisons and Tukey's range test was used for multiple comparisons; $^*p < 0.05$ and $^{**}p < 0.01$.

DATA AVAILABILITY

The data generated by this study are available on request from the corresponding author.

ACKNOWLEDGMENTS

The authors thank Tara Weisz at Duke University for her mouse husbandry work and Amy McArdle at Duke University for instruction ensuring the rigorous performance of mouse tail vein injection and submandibular bleeding technique. We also thank Dr. Laura Barisoni for her expertise in and guidance on the assessment of renal pathology. This work was supported by grants from the National Institutes of Health (National Eye Institute R01 EY031748 [C.B.R.], P30 EY005722 [Duke Eye Center]), Foundation Fighting Blindness awards (BR-CMM-0615-0681-DUKE and BR-CMM-0522-0830-DUKE [C.B.R.]), and an unrestricted grant from Research to Prevent Blindness (Duke Eye Center). The figures were created with support from BioRender.

AUTHOR CONTRIBUTIONS

Conceptualization, C.B.R., L.A.C., and D.G. Investigation, L.A.C., D.G., C.G.H., C.R.S., T.R.L., J.A., E.J.F., and M.V. Data curation, C.B.R., L.A.C., and J.A. Formal analysis, L.A.C., J.A., and W.K.M. Methodology, C.B.R., J.G.F., L.A.C., and D.G. Project administration, C.B.R. and L.A.C. Resources, C.B.R. and J.G.F. Validation, L.A.C., W.K.M., and C.G.H. Visualization, L.A.C., W.K.M., and C.B.R. Funding acquisition, C.B.R. Writing – original draft, L.A.C. and C.B.R. Writing – review & editing, L.A.C., T.R.L., W.K.M., and C.B.R.

DECLARATION OF INTERESTS

C.B.R., Applied Genetic Technologies Corporation (F [sponsored research funding]), Aevitas Therapeutics (F), and 4DMT (C [consultant]).

SUPPLEMENTAL INFORMATION

Supplemental information can be found online at https://doi.org/10.1016/j.ymthe.2025.04.035.

REFERENCES

1. Wong, C.W., Wong, T.Y., Cheng, C.Y., and Sabanayagam, C. (2014). Kidney and eye diseases: common risk factors, etiological mechanisms, and pathways. Kidney Int. *85*, 1290–1302. https://doi.org/10.1038/KI.2013.491.

2. Farrah, T.E., Dhillon, B., Keane, P.A., Webb, D.J., and Dhaun, N. (2020). The eye, the kidney, and cardiovascular disease: old concepts, better tools, and new horizons. Kidney Int. *98*, 323–342. https://doi.org/10.1016/J.KINT.2020.01.039.

3. GBD 2019 Blindness and Vision Impairment Collaborators; Vision Loss Expert Group of the Global Burden of Disease Study (2021). Causes of blindness and vision impairment in 2020 and trends over 30 years, and prevalence of avoidable blindness in relation to VISION 2020: the Right to Sight: an analysis for the Global Burden of Disease Study. Lancet Glob. Health *9*, e144–e160. https://doi.org/10.1016/S2214-109X(20)30489-7.

4. Rein, D.B., Wittenborn, J.S., Burke-Conte, Z., Gulia, R., Robalik, T., Ehrlich, J.R., Lundeen, E.A., and Flaxman, A.D. (2022). Prevalence of Age-Related Macular Degeneration in the US in 2019. JAMA Ophthalmol. *140*, 1202–1208. https://doi.org/10.1001/JAMAOPHTHALMOL.2022.4401.

5. Cameron, J.S., Turner, D.R., Heaton, J., Williams, D.G., Ogg, C.S., Chantler, C., Haycock, G.B., and Hicks, J. (1983). Idiopathic mesangiocapillary glomerulonephritis. Comparison of types I and II in children and adults and long-term prognosis. Am. J. Med. *74*, 175–192. https://doi.org/10.1016/0002-9343(83)90606-X.

6. Boyer, O., Noël, L.H., Balzamo, E., Guest, G., Biebuyck, N., Charbit, M., Salomon, R., Frémeaux-Bacchi, V., and Niaudet, P. (2008). Complement factor H deficiency and posttransplantation glomerulonephritis with isolated C3 deposits. Am. J. Kidney Dis. *51*, 671–677. https://doi.org/10.1053/J.AJKD.2007.11.032.

7. Boon, C.J.F., van de Kar, N.C., Klevering, B.J., Keunen, J.E.E., Cremers, F.P.M., Klaver, C.C.W., Hoyng, C.B., Daha, M.R., and den Hollander, A.I. (2009). The spectrum of phenotypes caused by variants in the CFH gene. Mol. Immunol. *46*, 1573–1594. https://doi.org/10.1016/J.MOLIMM.2009.02.013.

8. Renner, B., Laskowski, J., Poppelaars, F., Ferreira, V.P., Blaine, J., Antonioli, A.H., Hannan, J.P., Kovacs, J.M., van Kooten, C., You, Z., et al. (2022). Factor H related proteins modulate complement activation on kidney cells. Kidney Int. *102*, 1331–1344. https://doi.org/10.1016/J.KINT.2022.07.035.

9. Sethi, S., Fervenza, F.C., Zhang, Y., Zand, L., Vrana, J.A., Nasr, S.H., Theis, J.D., Dogan, A., and Smith, R.J.H. (2012). C3 glomerulonephritis: clinicopathological findings, complement abnormalities, glomerular proteomic profile, treatment, and follow-up. Kidney Int. *82*, 465–473. https://doi.org/10.1038/KI.2012.212.

10. Barbour, T.D., Pickering, M.C., and Terence Cook, H. (2013). Dense deposit disease and C3 glomerulopathy. Semin. Nephrol. *33*, 493–507. https://doi.org/10.1016/J.SEMNEPHROL.2013.08.002.

11. Savige, J., Amos, L., Ierino, F., Mack, H.G., Symons, R.C.A., Hughes, P., Nicholls, K., and Colville, D. (2016). Retinal disease in the C3 glomerulopathies and the risk of impaired vision. Ophthalmic Genet. *37*, 369–376. https://doi.org/10.3109/13816810.2015.1101777.

12. D'souza, Y.B., Jones, C.J.P., Short, C.D., Roberts, I.S.D., and Bonshek, R.E. (2009). Oligosaccharide composition is similar in drusen and dense deposits in membranoproliferative glomerulonephritis type II. Kidney Int. *75*, 824–827. https://doi.org/10.1038/KI.2008.658.

13. Mullins, R.F., Aptsiauri, N., and Hageman, G.S. (2001). Structure and composition of drusen associated with glomerulonephritis: implications for the role of complement activation in drusen biogenesis. Eye (Lond) *15*, 390–395. https://doi.org/10.1038/EYE.2001.142.

14. Li, M., Atmaca-Sonmez, P., Othman, M., Branham, K.E.H., Khanna, R., Wade, M.S., Li, Y., Liang, L., Zareparsi, S., Swaroop, A., and Abecasis, G.R. (2006). CFH haplotypes without the Y402H coding variant show strong association with susceptibility to age-related macular degeneration. Nat. Genet. *38*, 1049–1054. https://doi.org/10.1038/NG1871.

15. Raychaudhuri, S., Iartchouk, O., Chin, K., Tan, P.L., Tai, A.K., Ripke, S., Gowrisankar, S., Vemuri, S., Montgomery, K., Yu, Y., et al. (2011). A rare penetrant mutation in CFH confers high risk of age-related macular degeneration. Nat. Genet. *43*, 1232–1236. https://doi.org/10.1038/NG.976.

16. Flaxel, C.J., Adelman, R.A., Bailey, S.T., Fawzi, A., Lim, J.I., Vemulakonda, G.A., and Ying, G.S. (2020). Age-Related Macular Degeneration Preferred Practice Pattern®. Ophthalmology *127*, P1–P65. https://doi.org/10.1016/J.OPHTHA.2019.09.024.

17. De Jong, P.T.V.M. (2018). Elusive drusen and changing terminology of AMD. Eye *32*, 904–914. https://doi.org/10.1038/eye.2017.298.

18. Klein, M.L., Ferris, F.L., Armstrong, J., Hwang, T.S., Chew, E.Y., Bressler, S.B., and Chandra, S.R.; AREDS Research Group (2008). Retinal precursors and the development of geographic atrophy in age-related macular degeneration. Ophthalmology *115*, 1026–1031. https://doi.org/10.1016/J.OPHTHA.2007.08.030.

19. Klein, R.J., Zeiss, C., Chew, E.Y., Tsai, J.Y., Sackler, R.S., Haynes, C., Henning, A.K., SanGiovanni, J.P., Mane, S.M., Mayne, S.T., et al. (2005). Complement factor H polymorphism in age-related macular degeneration. Science *308*, 385–389. https://doi.org/10.1126/SCIENCE.1109557.

20. Hageman, G.S., Anderson, D.H., Johnson, L.V., Hancox, L.S., Taiber, A.J., Hardisty, L.I., Hageman, J.L., Stockman, H.A., Borchardt, J.D., Gehrs, K.M., et al. (2005). A common haplotype in the complement regulatory gene factor H (HF1/CFH) predisposes individuals to age-related macular degeneration. Proc. Natl. Acad. Sci. USA *102*, 7227–7232. https://doi.org/10.1073/PNAS.0501536102.

21. Haines, J.L., Hauser, M.A., Schmidt, S., Scott, W.K., Olson, L.M., Gallins, P., Spencer, K.L., Kwan, S.Y., Noureddine, M., Gilbert, J.R., et al. (2005). Complement factor H variant increases the risk of age-related macular degeneration. Science *308*, 419–421. https://doi.org/10.1126/SCIENCE.1110359.

22. Edwards, A.O., Ritter, R., Abel, K.J., Manning, A., Panhuysen, C., and Farrer, L.A. (2005). Complement factor H polymorphism and age-related macular degeneration. Science *308*, 421–424. https://doi.org/10.1126/SCIENCE.1110189.

23. Pappas, C.M., Zouache, M.A., Matthews, S., Faust, C.D., Hageman, J.L., Williams, B. L., Richards, B.T., and Hageman, G.S. (2021). Protective chromosome 1q32 haplotypes mitigate risk for age-related macular degeneration associated with the CFH-CFHR5 and ARMS2/HTRA1 loci. Hum. Genomics *15*, 60. https://doi.org/10.1186/S40246-021-00359-8.

24. Pickering, M.C., Cook, H.T., Warren, J., Bygrave, A.E., Moss, J., Walport, M.J., and Botto, M. (2002). Uncontrolled C3 activation causes membranoproliferative glomerulonephritis in mice deficient in complement factor H. Nat. Genet. *31*, 424–428. https://doi.org/10.1038/NG912.

25. Piras, R., Breno, M., Valoti, E., Alberti, M., Iatropoulos, P., Mele, C., Bresin, E., Donadelli, R., Cuccarolo, P., Smith, R.J.H., et al. (2021). CFH and CFHR Copy Number Variations in C3 Glomerulopathy and Immune Complex-Mediated Membranoproliferative Glomerulonephritis. Front. Genet. *12*, 670727. https://doi.org/10.3389/FGENE.2021.670727.

26. Grigsby, D., Klingeborn, M., Kelly, U., Chew, L.A., Asokan, A., Devlin, G., Smith, S., Keyes, L., Timmers, A., Scaria, A., and Bowes Rickman, C. (2023). AAV Gene Augmentation of Truncated Complement Factor H Differentially Rescues Ocular Complement Dysregulation in a Mouse Model. Invest. Ophthalmol. Vis. Sci. *64*, 25. https://doi.org/10.1167/IOVS.64.10.25.

27. Clark, S.J., Keenan, T.D.L., Fielder, H.L., Collinson, L.J., Holley, R.J., Merry, C.L.R., van Kuppevelt, T.H., Day, A.J., and Bishop, P.N. (2011). Mapping the differential distribution of glycosaminoglycans in the adult human retina, choroid, and sclera. Invest. Ophthalmol. Vis. Sci. *52*, 6511–6521. https://doi.org/10.1167/IOVS.11-7909.

28. Herbert, A.P., Deakin, J.A., Schmidt, C.Q., Blaum, B.S., Egan, C., Ferreira, V.P., Pangburn, M.K., Lyon, M., Uhrín, D., and Barlow, P.N. (2007). Structure shows that a glycosaminoglycan and protein recognition site in factor H is perturbed by age-related macular degeneration-linked single nucleotide polymorphism. J. Biol. Chem. *282*, 18960–18968. https://doi.org/10.1074/JBC.M609636200.

29. Ding, J.D., Kelly, U., Landowski, M., Toomey, C.B., Groelle, M., Miller, C., Smith, S. G., Klingeborn, M., Singhapricha, T., Jiang, H., et al. (2015). Expression of human complement factor H prevents age-related macular degeneration-like retina damage and kidney abnormalities in aged Cfh knockout mice. Am. J. Pathol. *185*, 29–42. https://doi.org/10.1016/J.AJPATH.2014.08.026.

30. Hajishengallis, G., Reis, E.S., Mastellos, D.C., Ricklin, D., and Lambris, J.D. (2017). Novel mechanisms and functions of complement. Nat. Immunol. *18*, 1288–1298. https://doi.org/10.1038/NI.3858.

31. Toomey, C.B., Kelly, U., Saban, D.R., and Bowes Rickman, C. (2015). Regulation of age-related macular degeneration-like pathology by complement factor H. Proc. Natl. Acad. Sci. USA *112*, E3040–E3049. https://doi.org/10.1073/PNAS.1424391112.

32. Fakhouri, F., De Jorge, E.G., Brune, F., Azam, P., Cook, H.T., and Pickering, M.C. (2010). Treatment with human complement factor H rapidly reverses renal complement deposition in factor H-deficient mice. Kidney Int. *78*, 279–286. https://doi.org/10.1038/KI.2010.132.

33. Landowski, M., Kelly, U., Klingeborn, M., Groelle, M., Ding, J.D., Grigsby, D., and Bowes Rickman, C. (2019). Human complement factor H Y402H polymorphism causes an age-related macular degeneration phenotype and lipoprotein dysregulation in mice. Proc. Natl. Acad. Sci. USA *116*, 3703–3711. https://doi.org/10.1073/PNAS.1814014116.

34. D'Agati, V.D., and Bomback, A.S. (2012). C3 glomerulopathy: what's in a name? Kidney Int. *82*, 379–381. https://doi.org/10.1038/KI.2012.80.

35. Smith, R.J.H., Appel, G.B., Blom, A.M., Cook, H.T., D'Agati, V.D., Fakhouri, F., Fremeaux-Bacchi, V., Józsi, M., Kavanagh, D., Lambris, J.D., et al. (2019). C3 glomerulopathy — understanding a rare complement-driven renal disease. Nat. Rev. Nephrol. *15*, 129–143. https://doi.org/10.1038/s41581-018-0107-2.

36. Friese, M.A., Hellwage, J., Jokiranta, T.S., Meri, S., Peter, H.H., Eibel, H., and Zipfel, P.F. (1999). FHL-1/reconectin and factor H: two human complement regulators which are encoded by the same gene are differently expressed and regulated. Mol. Immunol. *36*, 809–818. https://doi.org/10.1016/S0161-5890(99)00101-7.

37. Zincarelli, C., Soltys, S., Rengo, G., and Rabinowitz, J.E. (2008). Analysis of AAV serotypes 1-9 mediated gene expression and tropism in mice after systemic injection. Mol. Ther. *16*, 1073–1080. https://doi.org/10.1038/MT.2008.76.

38. Bell, P., Gao, G., Haskins, M.E., Wang, L., Sleeper, M., Wang, H., Calcedo, R., Vandenberghe, L.H., Chen, S.J., Weisse, C., et al. (2011). Evaluation of adeno-associated viral vectors for liver-directed gene transfer in dogs. Hum. Gene Ther. *22*, 985–997. https://doi.org/10.1089/HUM.2010.194.

39. Chen, S.J., Sanmiguel, J., Lock, M., McMenamin, D., Draper, C., Limberis, M.P., Kassim, S.H., Somanathan, S., Bell, P., Johnston, J.C., et al. (2013). Biodistribution of AAV8 vectors expressing human low-density lipoprotein receptor in a mouse model of homozygous familial hypercholesterolemia. Hum. Gene Ther. Clin. Dev. *24*, 154–160. https://doi.org/10.1089/HUMC.2013.082.

40. Wu, Z., Yang, H., and Colosi, P. (2010). Effect of genome size on AAV vector packaging. Mol. Ther. *18*, 80–86. https://doi.org/10.1038/MT.2009.255.

41. Paixão-Cavalcante, D., Hanson, S., Botto, M., Cook, H.T., and Pickering, M.C. (2009). Factor H facilitates the clearance of GBM bound iC3b by controlling C3 activation in fluid phase. Mol. Immunol. *46*, 1942–1950. https://doi.org/10.1016/J.MOLIMM.2009.03.030.

42. Martínez-Barricarte, R., Heurich, M., Valdes-Cañedo, F., Vazquez-Martul, E., Torreira, E., Montes, T., Tortajada, A., Pinto, S., Lopez-Trascasa, M., Morgan, B. P., et al. (2010). Human C3 mutation reveals a mechanism of dense deposit disease pathogenesis and provides insights into complement activation and regulation. J. Clin. Invest. *120*, 3702–3712. https://doi.org/10.1172/JCI43343.

43. Chauvet, S., Roumenina, L.T., Bruneau, S., Marinozzi, M.C., Rybkine, T., Schramm, E.C., Java, A., Atkinson, J.P., Aldigier, J.C., Bridoux, F., et al. (2016). A Familial C3GN Secondary to Defective C3 Regulation by Complement Receptor 1 and Complement Factor H. J. Am. Soc. Nephrol. *27*, 1665–1677. https://doi.org/10.1681/ASN.2015040348.

44. Levy, M., Halbwachs-Mecarelli, L., Gubler, M.C., Kohout, G., Bensenouci, A., Niaudet, P., Hauptmann, G., and Lesavre, P. (1986). H deficiency in two brothers with atypical dense intramembranous deposit disease. Kidney Int. *30*, 949–956. https://doi.org/10.1038/KI.1986.278.

45. Licht, C., Heinen, S., Józsi, M., Löschmann, I., Saunders, R.E., Perkins, S.J., Waldherr, R., Skerka, C., Kirschfink, M., Hoppe, B., and Zipfel, P.F. (2006). Deletion of Lys224 in regulatory domain 4 of Factor H reveals a novel pathomechanism for dense deposit disease (MPGN II). Kidney Int. *70*, 42–50. https://doi.org/10.1038/SJ.KI.5000269.

46. Abrera-Abeleda, M.A., Nishimura, C., Smith, J.L.H., Sethi, S., McRae, J.L., Murphy, B.F., Silvestri, G., Skerka, C., Józsi, M., Zipfel, P.F., et al. (2006). Variations in the complement regulatory genes factor H (CFH) and factor H related 5 (CFHR5) are associated with membranoproliferative glomerulonephritis type II (dense deposit disease). J. Med. Genet. *43*, 582–589. https://doi.org/10.1136/JMG.2005.038315.

47. Keeler, G.D., Gaddie, C.D., Sagadevan, A.S., Senior, K.G., Côté, I., Rechdan, M., Min, D., Mahan, D., Poma, B., and Hoffman, B.E. (2023). Induction of antigen-specific tolerance by hepatic AAV immunotherapy regardless of T cell epitope usage or mouse strain background. Mol. Ther. Methods Clin. Dev. *28*, 177–189. https://doi.org/10.1016/J.OMTM.2022.12.011.

48. Keeler, G.D., Markusic, D.M., and Hoffman, B.E. (2019). Liver induced transgene tolerance with AAV vectors. Cell. Immunol. *342*, 103728. https://doi.org/10.1016/J.CELLIMM.2017.12.002.

49. Kerr, H., Wong, E., Makou, E., Yang, Y., Marchbank, K., Kavanagh, D., Richards, A., Herbert, A.P., and Barlow, P.N. (2017). Disease-linked mutations in factor H reveal pivotal role of cofactor activity in self-surface-selective regulation of complement activation. J. Biol. Chem. *292*, 13345–13360. https://doi.org/10.1074/JBC.M117.795088.

50. Pickering, M.C., de Jorge, E.G., Martinez-Barricarte, R., Recalde, S., Garcia-Layana, A., Rose, K.L., Moss, J., Walport, M.J., Cook, H.T., de Córdoba, S.R., and Botto, M. (2007). Spontaneous hemolytic uremic syndrome triggered by complement factor H lacking surface recognition domains. J. Exp. Med. *204*, 1249–1256. https://doi.org/10.1084/JEM.20070301.

51. Boutaud, A., Borza, D.B., Bondar, O., Gunwar, S., Netzer, K.O., Singh, N., Ninomiya, Y., Sado, Y., Noelken, M.E., and Hudson, B.G. (2000). Type IV collagen of the glomerular basement membrane. Evidence that the chain specificity of network assembly is encoded by the noncollagenous NC1 domains. J. Biol. Chem. *275*, 30716–30724. https://doi.org/10.1074/JBC.M004569200.

52. Booij, J.C., Baas, D.C., Beisekeeva, J., Gorgels, T.G.M.F., and Bergen, A.A.B. (2010). The dynamic nature of Bruch's membrane. Prog. Retin. Eye Res. *29*, 1–18. https://doi.org/10.1016/J.PRETEYERES.2009.08.003.

53. Kelly, U.L., Grigsby, D., Cady, M.A., Landowski, M., Skiba, N.P., Liu, J., Remaley, A. T., Klingeborn, M., and Bowes Rickman, C. (2020). High-density lipoproteins are a potential therapeutic target for age-related macular degeneration. J. Biol. Chem. *295*, 13601–13616. https://doi.org/10.1074/JBC.RA119.012305.

54. Rose, K.L., Paixao-Cavalcante, D., Fish, J., Manderson, A.P., Malik, T.H., Bygrave, A. E., Lin, T., Sacks, S.H., Walport, M.J., Cook, H.T., et al. (2008). Factor I is required for the development of membranoproliferative glomerulonephritis in factor H-deficient mice. J. Clin. Invest. *118*, 608–618. https://doi.org/10.1172/JCI32525.

55. Bhutto, I.A., Baba, T., Merges, C., Juriasinghani, V., McLeod, D.S., and Lutty, G.A. (2011). C-reactive protein and complement factor H in aged human eyes and eyes with age-related macular degeneration. Br. J. Ophthalmol. *95*, 1323–1330. https://doi.org/10.1136/BJO.2010.199216.

56. Flannery, J.G., and Visel, M. (2013). Adeno-associated viral vectors for gene therapy of inherited retinal degenerations. Methods Mol. Biol. *935*, 351–369. https://doi.org/10.1007/978-1-62703-080-9_25.

YMTHE, Volume *33*

Supplemental Information

Truncated complement factor H Y402 gene therapy rescues C3 glomerulonephritis

Lindsey A. Chew, Daniel Grigsby, C. Garren Hester, Joshua Amason, W. Kyle McPherson, Edward J. Flynn III, Meike Visel, Christopher R. Starr, John G. Flannery, Tylor R. Lewis, and Catherine Bowes Rickman

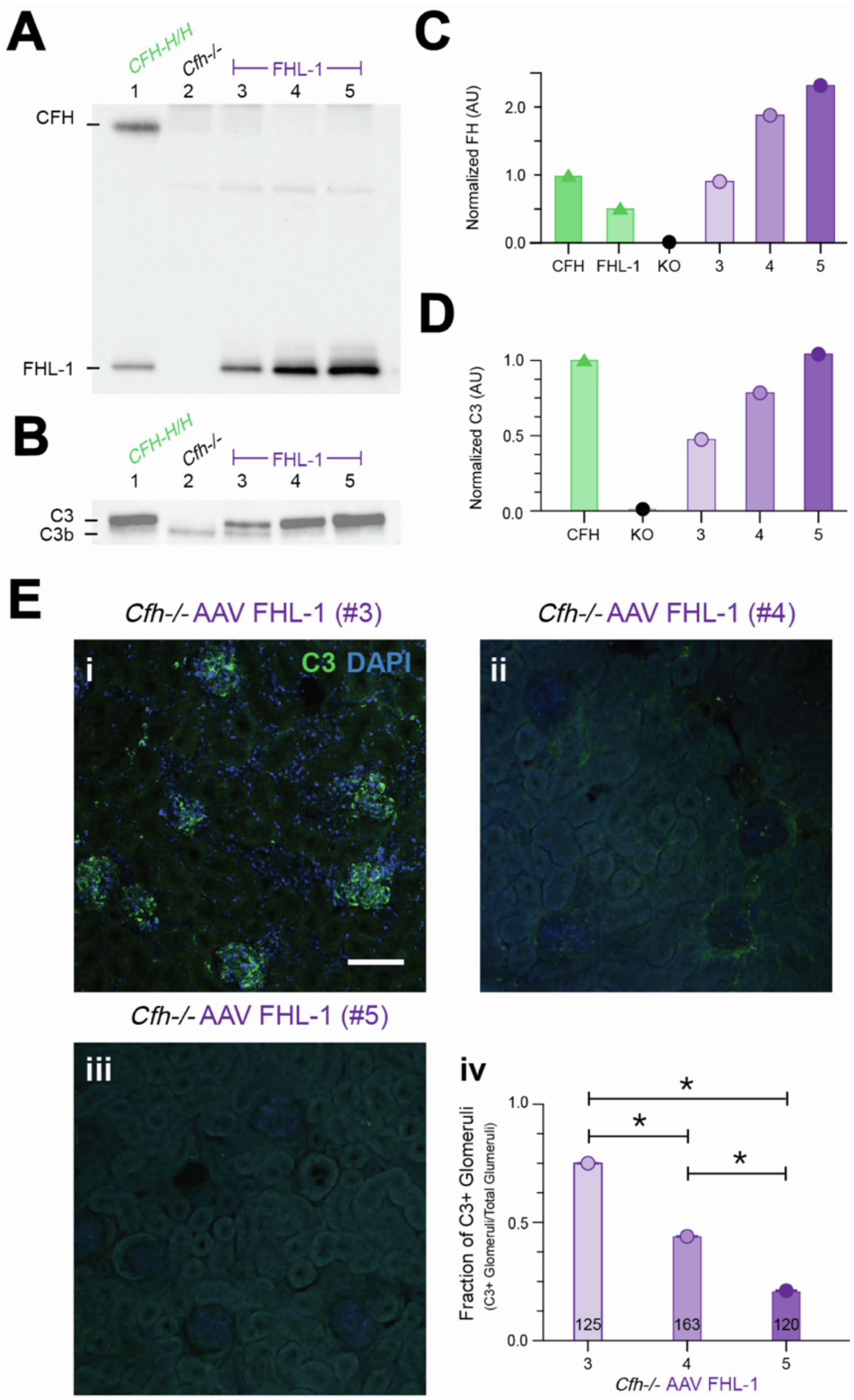

Figure S1. Concentration-dependent, AAV-mediated FHL-1 replacement reduces C3 deposit accumulation and resolves C3G.
(**A**) Western blot analysis of circulating plasma levels of FHL-1 in *Cfh-/-* mice following treatment with AAV-mediated FHL-1 replacement. Each lane shows the corresponding levels of tCFH for a single animal. Lane 1 is a positive control loaded with *CFH H/H* plasma and shows full-length CFH. Lane 2 is a negative control loaded with untreated *Cfh-/-* plasma. (**B**) Western blot analysis of circulating plasma levels of C3/C3b in corresponding *Cfh-/-* mice following treatment with AAV-mediated FHL-1 replacement. Lane 1 is a positive control loaded with *CFH H/H* plasma and shows C3/C3b. Lane 2 is a negative control loaded with untreated *Cfh-/-* plasma and shows C3b but no intact C3. (**C**) Densitometric analysis of Western blot (A) with bar graph (derived from panel A) showing normalized CFH expression in several *Cfh-/-* mice ± AAV-FHL-1 treatment. Normalized FH is calculated as a ratio of FHL-1 expression to endogenous expression of full-length CFH in the CFH-H/H (Lane 1). (**D**) Densitometric analysis of Western

blot with bar graph (derived from panel B) of normalized C3 for each animal and normalized against *CFH-H/H,* C3G-negative control mice. (**E**) Representative immunofluorescent confocal imaging of glomeruli from kidney sections of the corresponding mice (*i-iii*). Scale bar is 100 microns. (*iv*) Bar graph indicating the fraction of C3-positive (C3+) glomeruli out of the total glomeruli for which confocal imaging was obtained. The number of glomeruli analyzed for each cohort is shown at the base of each bar. Unpaired t-test was used for comparison between treatment cohorts, *$p<0.01$.
